# Melatonin Enhances Cisplatin and Radiation Cytotoxicity in Head and Neck Squamous Cell Carcinoma by Stimulating Mitochondrial ROS Generation, Apoptosis, and Autophagy

**DOI:** 10.1155/2019/7187128

**Published:** 2019-02-28

**Authors:** Beatriz I. Fernandez-Gil, Ana Guerra-Librero, Ying-Qiang Shen, Javier Florido, Laura Martínez-Ruiz, Sergio García-López, Christian Adan, César Rodríguez-Santana, Darío Acuña-Castroviejo, Alfredo Quiñones-Hinojosa, José Fernández-Martínez, Ahmed E. Abdel Moneim, Luis C. López, José M. Rodríguez Ferrer, Germaine Escames

**Affiliations:** ^1^Instituto de Biotecnología, Centro de Investigación Biomédica, Universidad de Granada, Granada, Spain; ^2^Departamento de Fisiología, Facultad de Medicina, Universidad de Granada, Granada, Spain; ^3^CIBERFES, Ibs.Granada, Hospital Campus de la Salud, 18016 Granada, Spain; ^4^Department of Neurosurgery, Mayo Clinic, School of Medicine, Jacksonville, Florida, USA; ^5^Department of Zoology and Entomology, Faculty of Science, Helwan University, Cairo, Egypt

## Abstract

Head and neck cancer is the sixth leading cancer by incidence worldwide. Unfortunately, drug resistance and relapse are the principal limitations of clinical oncology for many patients, and the failure of conventional treatments is an extremely demoralizing experience. It is therefore crucial to find new therapeutic targets and drugs to enhance the cytotoxic effects of conventional treatments without potentiating or offsetting the adverse effects. Melatonin has oncostatic effects, although the mechanisms involved and doses required remain unclear. The purpose of this study is to determine the precise underlying mitochondrial mechanisms of melatonin, which increase the cytotoxicity of oncological treatments, and also to propose new melatonin treatments in order to alleviate and reverse radio- and chemoresistant processes. We analyzed the effects of melatonin on head and neck squamous cell carcinoma (HNSCC) cell lines (Cal-27 and SCC-9), which were treated with 0.1, 0.5, 1, and 1.5 mM melatonin combined with 8 Gy irradiation or 10 *μ*M cisplatin. Clonogenic and MTT assays, as well as autophagy and apoptosis, involving flow cytometry and western blot, were performed in order to determine the cytotoxic effects of the treatments. Mitochondrial function was evaluated by measuring mitochondrial respiration, mtDNA content (RT-PCR), and mitochondrial mass (NAO). ROS production, antioxidant enzyme activity, and GSH/GSSG levels were analyzed using a fluorometric method. We show that high concentrations of melatonin potentiate the cytotoxic effects of radiotherapy and CDDP in HNSCC, which are associated with increased mitochondrial function in these cells. In HNSCC, melatonin induces intracellular ROS, whose accumulation plays an upstream role in mitochondria-mediated apoptosis and autophagy. Our findings indicate that melatonin, at high concentrations, combined with cisplatin and radiotherapy to improve its effectiveness, is a potential adjuvant agent.

## 1. Introduction

Head and neck cancer, which is the sixth leading cancer by incidence worldwide, with more than 300,000 mortalities annually [1], has become a major health burden, especially as cell resistance to radio and chemotherapy develops.

Radiotherapy (RT), which is one of the most commonly used tumor treatments [2], damages biomolecules, such as proteins and lipoids, particularly DNA, resulting in the termination of cell division and proliferation and even in cell necrosis or apoptosis. However, many unwanted effects, such as radioresistance, can complicate the prognosis [2]. One way to overcome these problems is to increase RT effectiveness by using radiosensitizers to enhance tumor cell radiosensitivity [3].

On the other hand, cisplatin (CDDP), one of the most commonly used chemotherapeutic agents, is the treatment of choice for most head and neck squamous cell carcinoma (HNSCC) patients. CDDP is a highly reactive molecule that binds to RNA, DNA, and proteins to form different types of adducts. CDDP also induces mitochondria-dependent reactive oxygen species (ROS) formation which contributes to cell-killing processes by enhancing the damaging effect of drugs on nuclear DNA (nDNA) [4]. However, the high incidence of chemoresistance and its many side effects limit the clinical usefulness of CDDP as an anticancer treatment [5, 6]. Therefore, new anticancer therapeutic strategies to attenuate cytotoxicity in normal tissues and to prevent or reverse the development of radio and chemoresistance are required.

Mitochondria have a major impact on cancer cells due to the source of ATP, their capacity to produce ROS, and their central position in the apoptosis signaling pathway [7, 8]. Although very low supraphysiological levels of mitochondrial ROS can promote tumor diversification by favoring mutagenesis [9], ROS overproduction, leading to severe mitochondrial dysfunction, is generally incompatible with tumor progression, which promotes cell death and cellular senescence [10]. Thus, given the impact of mitochondrial metabolism on the treatment response, a considerable effort has been devoted to developing a chemo/radiosensitization strategy involving the development of molecules to target mitochondria [9, 11]. However, one of the main drawbacks of this strategy of targeting mitochondria to kill malignant cells or to increase their sensitivity to treatment is that multiple immune effector cells bear a remarkable metabolic similarity to cancer cells [9, 12].

Although the indoleamine melatonin (N-acetyl-5-methoxytryptamine) is synthetized in the pineal gland, it is produced by many other organs at even higher concentrations [13]. Melatonin has a variety of biological features including anti-inflammatory and antioxidant activity, as well as immune system regulation mechanisms. It also has oncostatic effects, although the mechanisms involved remain unclear [14–17]. Our previous studies show that melatonin enhances the cytotoxic effects of rapamycin in HNSCC cells [18]. However, little data exist on the mechanisms of melatonin involved in increasing chemo- and radiotherapy-induced cancer cell injury or cell death and in simultaneously decreasing its adverse effects.

In this study, we first explore the potential capacity of melatonin to enhance the antitumor effects of irradiation and CDDP on HNSCC. We then investigate the precise underlying mitochondrial mechanisms which enhance the cytotoxic effects of these treatments on HNSCC.

## 2. Materials and Methods

### 2.1. Cell Culture

Human tongue squamous carcinoma cell lines Cal-27 and SCC-9, obtained from the American Type Culture Collection (ATCC® CRL2095™ and CRL1629™, respectively) in the Cell Bank of the Centre for Scientific Instrumentation at the University of Granada, were cultured in a humidified atmosphere (5% CO_2_ and 95% air at 37°C). The cells were cultured in high-glucose Dulbecco's modified Eagle's medium (DMEM), GlutaMAX supplemented with 10% fetal bovine serum, and 2% antibiotic-antimycotic (Fisher Scientific, Madrid, Spain) for Cal-27 and were grown in DMEM-F12 Nutrient Mixture Ham medium (1 : 1) containing 2 mM L-glutamine (Fisher Scientific, Madrid, Spain) and 0.5 mM sodium pyruvate supplemented with 10% FBS, 0.4 *μ*g/mL hydrocortisone (Sigma-Aldrich, Madrid, Spain), and 2% antibiotic-antimycotic for SCC-9.

Melatonin stock solution (Fagron Ibérica S.A.U., Terrasa, Spain) was prepared in 15% propylene glycol (PG) (VWR, Radnor, PA, USA). Cells were grown to 60%-70% confluence and serum starved for 24 hours. Then, cells were treated with and without melatonin (100, 500, 1000, and 1500 *μ*M). After 48 hours, cells were exposed to 8 Gy irradiation using a cesium-137 gamma radiation source (8 Gy/min) or treated with 10 *μ*M CDDP for 5 hours (Sigma-Aldrich, Madrid, Spain). Cells of the control group were treated with a vehicle (PG 15%). Assays were performed 48 hours after irradiation and CDDP treatment.

### 2.2. Colony Formation Assay

Cells were plated into 6-well plates and allowed to attach overnight. They were treated with melatonin and, after 48 hours, were irradiated or treated with CDDP. Colonies were allowed to grow for 2 weeks to form colonies of at least 50 cells. Finally, the medium was removed, the cells were fixed, and the colonies were then stained with 2.3% crystal violet and counted [18].

### 2.3. Cell Proliferation Assay

Cell viability was determined using an MTT assay (Life Technologies, Madrid, Spain) based on the reduction of yellow 3-(4,5-dimethylthiazol-2-yl)-2,5-diphenyltetrazolium bromide to purple formazan by mitochondrial dehydrogenases. Cells were plated into 96-well plates, and the assay was performed according to the manufacturer's instruction.

### 2.4. Apoptosis

Apoptosis was measured by flow cytometry using FITC Annexin V staining (Immunostep, Salamanca, Spain). Cells were treated as described above. Finally, cells were collected, washed with cold PBS, and then simultaneously stained with FITC-labeled annexin V and PI and analyzed by flow cytometry in a Becton Dickinson FACSCanto II cytometer (Madrid, Spain).

### 2.5. Western Blot Analysis

Protein extraction and western blot analyses were performed as described previously [19]. Bax (sc-526), Bcl-2 (sc-492), ATG12 C6 (sc-271688), and GAPDH (sc-32233) antibodies and a mouse anti-goat IgG-HRP secondary antibody (sc-2354) were purchased from Santa Cruz Biotechnology (Santa Cruz, CA, USA), NIX from Sigma-Aldrich (Madrid, Spain), and HRP goat anti-mouse IgG from BD Pharmingen™ (San Jose, CA, USA). The proteins were visualized using a Western Lightning Plus-ECL chemiluminescence kit (PerkinElmer, Billerica, MA, USA) according to the manufacturer's protocol. Images were analyzed using the Kodak Image Station 2000R (Eastman Kodak Company, Rochester, NY, USA). Protein band intensity was normalized to GAPDH, and data were expressed in percentage terms relative to controls.

### 2.6. Mitochondrial Respiration

The oxygen consumption rate (OCR), an indicator of mitochondrial respiration under typical *in vitro* cell culture conditions [18], was determined using the Seahorse Extracellular Flux (XFe24) analyzer (Seahorse Bioscience, MA, USA). The day before the experiment, live treated cells (exclusion by trypan blue) were seeded in DMEM in 24-well culture plates at a density of 8 · 10^4^ cells/well and were allowed to adhere overnight in a cell culture incubator in order to minimize division or death. Cell growth and health were monitored using a microscope following the manufacturer's instructions, and the assay was only performed if the cells under all conditions formed a consistent monolayer. Subsequently, the assays were initiated by replacing the media with assay medium (Seahorse Bioscience), and the cells were equilibrated for 1 h at 37°C without CO_2_. The microplate was then placed into the XFe24 instrument to measure the OCR and free protons in the medium. Basal OCR was measured three times and plotted as a function of cells under the basal condition, followed by the sequential addition of oligomycin 1 mM. Subsequently, carbonyl cyanide 4-(trifluoromethoxy)phenylhydrazone (FCCP) 0.5 mM was added in two injections (1 mM in total). Finally, rotenone/antimycin A (1 mM) was injected. OCR was measured throughout the different injections of the test compounds. The progress curve was annotated to show the relative contribution of basal, ATP-linked, and maximal oxygen consumption after the addition of FCCP, and the reserve capacity of the cells. OCR values were normalized to cell number.

### 2.7. Determination of Mitochondrial Mass

We measured mitochondrial mass using acridine orange 10-nonyl bromide (NAO; Invitrogen Life Technologies, Madrid, Spain), which specifically binds to cardiolipin at the inner mitochondrial membrane, according to the protocol described by Shen et al. [18]. Fluorescence was read by an FLx800 microplate fluorescence reader (BioTek Instruments Inc., Winooski, VT, USA) at excitation 485 nm and emission 530 nm.

### 2.8. Mitochondrial DNA Quantification

Human mitochondrial DNA (mtDNA) was quantified by real-time PCR using the Stratagene Mx3005P Real-Time PCR System (Agilent Technologies Inc., CA, USA). We used primers and probes for the human 12S gene (mtDNA) and 18S. The mtDNA values were normalized to nDNA data (mtDNA/nDNA ratio).

### 2.9. Measurement of ROS Production

ROS production was measured using the 2′-7′-dichlorofluorescin diacetate (DCFH-DA) probe (Sigma-Aldrich, Madrid, Spain). Cells were seeded in 96-well culture plates. Then, the cells were incubated with 100 *μ*M DCFH-DA in culture medium without phenol red for 30 min at 37°C and then rinsed with PBS and filled with Krebs-Ringer bicarbonate buffer. ROS levels were measured in a multiwell plate reader spectrofluorometer (BioTek Instruments Inc., Winooski, VT, USA) for 45 minutes each 5 minutes at 485 nm to excitation and 530 nm to emission [18].

### 2.10. Measurement of GSH and GSSG Levels and GPx Activity

To measure glutathione (GSH) and glutathione disulfide (GSSG), we used an established fluorometric method using a microplate fluorescence reader (PowerWaveX FLx800; BioTek Instruments Inc., Winooski, VT) [20]. We spectrophotometrically measured glutathione peroxidase (GPx) activity in a UV spectrophotometer (model UV-1603; Shimadzu Deutschland GmBH, Duisburg, Germany) [21].

### 2.11. Statistical Analysis

Statistical analyses were performed using GraphPad Prism 6 scientific software (GraphPad Software Inc., La Jolla, CA) and one-way analysis of variance (ANOVA) followed by Tukey's multiple comparison tests. Data were expressed as the mean ± SEM of a minimum of three independent experiments. A *P* value of <.05 was considered statistically significant.

## 3. Results

### 3.1. Melatonin Enhances the Cytotoxic Effects of Irradiation and CDDP in HNSCC

**Figure 1:**
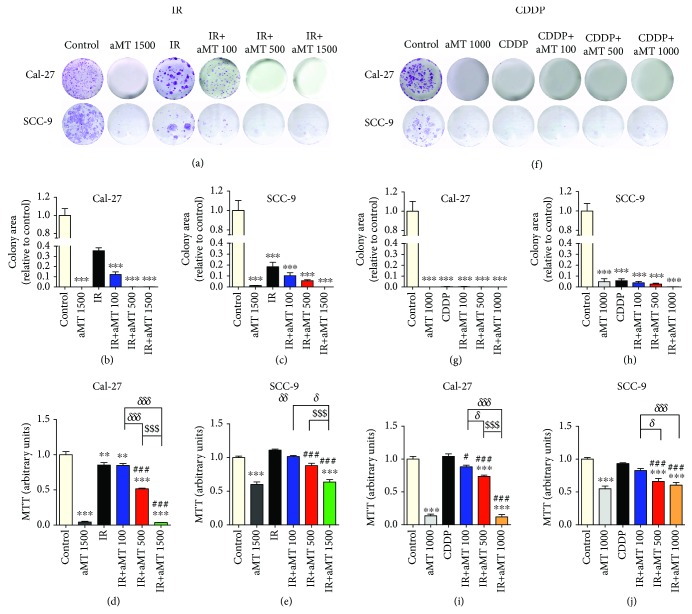
Melatonin increases the cytotoxic effects of irradiation (IR) and CDDP in HNSCC cell lines Cal-27 and SCC-9. Clonogenic assay of cells exposed to IR (a–c) or CDDP (f–h) and viability of cells exposed to IR (d, e) or CDDP (i, j). Treatment groups include the control (vehicle), IR (8 Gy), CDDP 10 *μ*M, melatonin (aMT) 1000 or 1500 *μ*M, and CDDP or IR plus aMT 100, 500, 1000, or 1500 *μ*M. *n* = 6 per group. Data are presented as mean ± SEM. ^∗∗^*P* < .01 and ^∗∗∗^*P*<.001 vs. the control, ^#^*P* < .05 and ^###^*P* < .001 vs. the IR- or CDDP-treated group, *^δ^P* < .05 and *^δδδ^P* < .001 vs. IR+aMT 100, and ^$^*P* < .05 and ^$$$^*P* < .001 vs. IR+aMT 500.

To evaluate the biological effect of melatonin on HNSCC sensitivity to irradiation and CDDP treatments, the clonogenic capacity and viability of both Cal-27 and SCC-9 were analyzed. As shown in Figures 1(a)–1(c), treatment with melatonin alone and in combination with irradiation significantly inhibited colony formation and resulted in a notable decrease in the colony ratio in a dose-dependent manner as compared to control or to irradiation alone. In fact, melatonin alone totally blocked colony growth. However, CDDP displayed a greater capacity than irradiation to decrease clonogenic formation (Figures 1(f)–1(h)).

MTT assays of both cell lines were also performed. In line with the inhibition of clonogenic capacity, melatonin markedly decreased cell viability in the irradiated cells in a dose-dependent manner, especially at doses 500 and 1500 *μ*M, as compared to control and irradiation alone (Figures 1(d)–1(e)), although SCC-9 cells were found to be more resistant than Cal-27 cells to the treatments. Surprisingly, 100 *μ*M melatonin did not significantly reduce viability, particularly in SCC-9 (Figures 1(d)–1(e)). On the other hand, melatonin significantly decreased cell viability in the CDDP-treated cells in a dose-dependent manner as compared to the control and CDDP alone (Figures 1(i)–1(j)). SCC-9 cells were also more resistant to melatonin exposure than Cal-27 cells. The results were more significant for Cal-27 cells, which were therefore used in subsequent experiments.

### 3.2. Melatonin Enhances the Apoptotic Effects of Irradiation and CDDP in HNSCC

**Figure 2:**
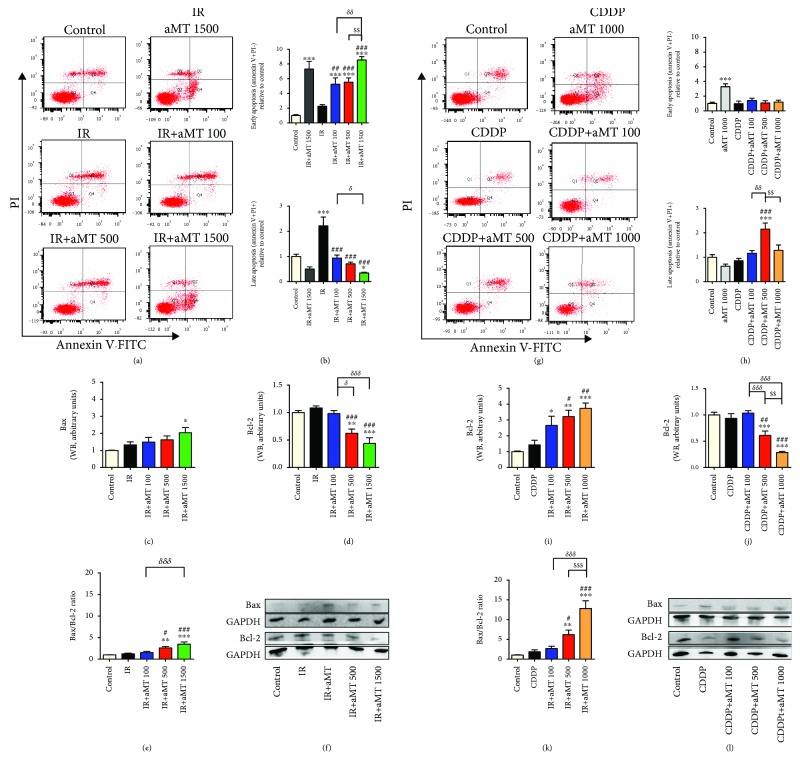
Combined treatment with melatonin and IR or CDDP increases apoptotic cell death in the HNSCC cell line Cal-27. Apoptosis was analyzed by flow cytometry. (a–g) Representative plots showing the redistribution of phosphatidylserine (annexin V staining) in the presence of propidium iodide (PI). The bottom right quadrant represents the percentage of early apoptotic cells (annexin V+/PI-), whereas the top right quadrant represents the percentage of late apoptotic cells (annexin V+/PI+). Statistical analysis of early and late apoptosis of cells exposed to IR (b) and CDDP (h), respectively. Western blot analysis (f–l) and densitometric quantification of Bax (c–i) and Bcl-2 (d–j) and the Bax/Bcl-2 ratio (e–k) in cells exposed to IR or CDDP, respectively. Treatment groups include the control (vehicle), IR (8 Gy), CDDP 10 *μ*M, melatonin (aMT) 1000 or 1500 *μ*M, and CDDP or IR plus aMT 100, 500, 1000, or 1500 *μ*M. *n* = 6 per group. Data are presented as mean ± SEM. ^∗^*P*<.05, ^∗∗^*P* < .01, and ^∗∗∗^*P*<.001 vs. the control; ^#^*P*<.05, ^##^*P*<.01, and ^###^*P*<.001 vs. the IR- or CDDP-treated group; *^δ^P*<.05, *^δδ^P*<.01, and *^δδδ^P*<.001 vs. IR+aMT 100; and ^$^*P*<.05, ^$$^*P*<.01, and ^$$$^*P*<.001 vs. IR+aMT 500.

Since the MTT assay is a quantitative measure of cell proliferation and a decrease in proliferating cells can be caused by either cell death or halted/slow proliferation, apoptotic cell death was therefore evaluated. Early apoptotic cells showed an annexin V-FITC+/PI- staining pattern, while late apoptotic cells exhibited an annexin V-FITC+/PI+ pattern (Figure 2) due to plasma membrane integrity loss [22]. In the combined melatonin/irradiation treatment, melatonin increased early apoptosis, which reached a maximum level at 1500 *μ*M as compared to that of the control (Figures 2(a)–2(b)). However, irradiation alone enhanced late apoptosis but did not affect early apoptosis (Figures 2(a)–2(b)). This indicates that melatonin increases the acute cytotoxicity of irradiation. By contrast, treatment with CDDP alone did not increase apoptosis pathway activation (Figures 2(g)–2(h)), while the combined treatment increased late apoptosis at a melatonin dose of 500 *μ*M (Figure 2(h)).

Apoptosis initiation is associated with the translocation of the inactive form of Bax from the cytoplasm to the mitochondria and suppression of the prosurvival protein Bcl-2. Bax and Bcl-2 protein expression was explored using western blot analysis. In line with the results above, Bcl-2 levels were clearly attenuated by 500 and 1500 *μ*M melatonin doses combined with irradiation, which increased the Bax/Bcl-2 ratio, with a maximum effect being observed at 1500 *μ*M (Figures 2(c)–2(f)). Moreover, melatonin combined with CDDP increased the Bax/Bcl-2 ratio more than when combined with irradiation despite using a lower concentration of melatonin (1000 *μ*M vs. 1500 *μ*M) (Figures 2(i)–2(l)). These data indicate that melatonin combined with CDDP increases cytotoxicity more than irradiation.

### 3.3. Enhancement of Mitochondrial Changes by Melatonin

**Figure 3:**
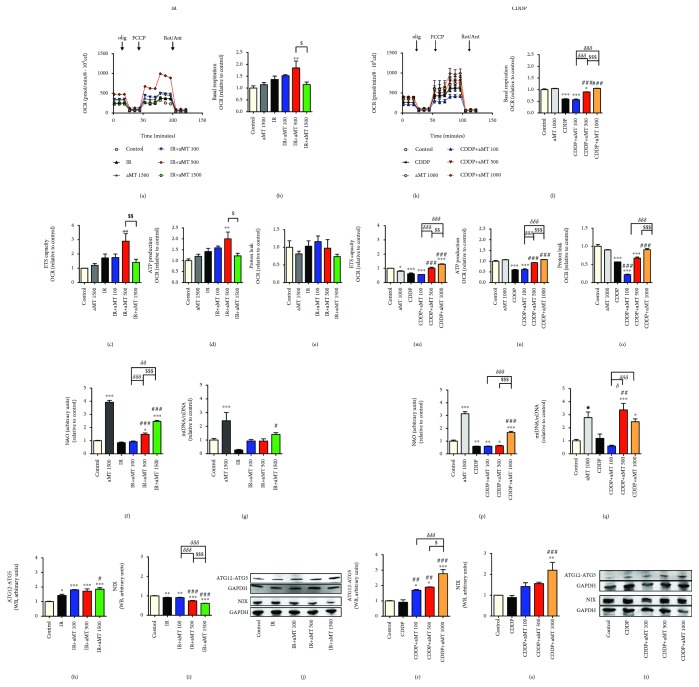
Effects of combined treatment with melatonin and IR or CDDP on Cal-27 HNSCC mitochondria. Oxygen consumption rate (OCR) (a, k), basal respiration (b, l), maximal respiratory capacity (ETS) (c, m), ATP production (d, n), and proton leak (e, o) in cells exposed to IR or CDDP, respectively. Mitochondrial mass (NAO) (f, p), mtDNA (g, q), western blot analysis, and densitometric quantification of ATG12-ATG5 (h, j, r, t) and NIX (i, j, s, t) in cells exposed to IR or CDDP, respectively. Treatment groups include the control (vehicle), IR (8 Gy), CDDP 10 *μ*M, melatonin (aMT) 1000 or 1500 *μ*M, and CDDP or IR plus aMT 100, 500, 1000, or 1500 *μ*M. *n* = 6 per group. Data are presented as mean ± SEM. ^∗^*P*<.05, ^∗∗^*P*<.01, and ^∗∗∗^*P*<.001 vs. the control; ^#^*P*<.05, ^##^*P*<.01, and ^###^*P*<.001 vs. the IR- or CDDP-treated group, *^δ^P*<.05, *^δδ^P*<.01, and *^δδδ^P*<.001 vs. IR+aMT 100; ^$^*P*<.05, ^$$^*P*<.01, and ^$$$^*P*<.001 vs. IR+aMT 500.

Mitochondria are critically involved in controlling regulated cell death triggered by different cancer treatments [9], and several metabolic aspects of the mitochondrial biology also influence therapeutic responses [23]. As melatonin regulates mitochondrial homeostasis [24, 25], we hypothesized that melatonin potentiates the cytotoxicity of irradiation and CDDP treatments modifying mitochondrial function. We first determined the oxygen consumption rate (OCR), which is an indicator of mitochondrial oxidative phosphorylation activity and ATP production. While measuring oxygen consumption rates, we sequentially added oligomycin, FCCP, and a combination of rotenone and antimycin to the cells to assess electron transport chain integrity (Figures 3(a)–3(k)). Irradiated Cal-27 cells exhibited a significant increase in basal respiration (Figure 3(b)) and in the maximal respiratory capacity of the electron transport system (ETS) (Figure 3(c)) at melatonin 500 *μ*M, which correlated with an increase of ATP (Figure 3(d)) with no change in proton leak (Figure 3(e)). Surprisingly, melatonin 1500 *μ*M caused a decrease in the OCR as compared to melatonin 500 *μ*M (Figures 3(b)–3(d)), suggesting defective mitochondrial function at higher concentrations of melatonin. By contrast, cells treated with CDDP alone or melatonin 100 *μ*M showed a decrease in the OCR (Figures 3(l)–3(n)), while melatonin doses of 500 and 1000 *μ*M rescued the OCR as compared to the control. These results are in line with the direct effect of CDDP on mitochondria which interferes with mtDNA transcription, resulting in reduced mitochondrial function [26].

To further determine changes in mitochondria in the presence of melatonin, we next analyzed mitochondrial mass and mtDNA (Figures 3(f), 3(g), 3(p), and 3(q)). To examine mitochondrial mass, we used acridine orange. Fluorescence data revealed that high concentrations of melatonin significantly augmented mitochondrial mass compared to the control, especially at 1500 and 1000 *μ*M, in cells exposed to irradiation or CDDP (Figures 3(f)–3(p)). Moreover, similar doses of melatonin increased the mtDNA/nDNA ratio (Figures 3(g) and 3(q)), indicating that melatonin significantly increases mtDNA.

In a previous study, we demonstrated that melatonin may induce both apoptosis and autophagy in HNSCC [18]. We determined the levels of autophagy-related proteins ATG12-ATG5, as autophagy requires the covalent attachment of protein Atg12 to protein ATG5 through an ubiquitin-like conjugation system and the mitophagic marker NIX. The combined melatonin and irradiation treatment increased ATG12-ATG5 levels at all doses (Figures 3(h) and 3(j)) but decreased NIX levels (Figures 3(i) and 3(j)). However, the combination of melatonin and CDDP increased both ATG12-ATG5 and NIX (Figures 3(r)–3(t)). These data indicate that the combined melatonin and CDDP treatment, with its higher toxicity, results in autophagy and mitophagy.

These results could denote a correlation between an increase of mitochondrial activity induced by melatonin and ROS production.

### 3.4. Enhancement of Oxidative Stress in the Presence of Melatonin

**Figure 4:**
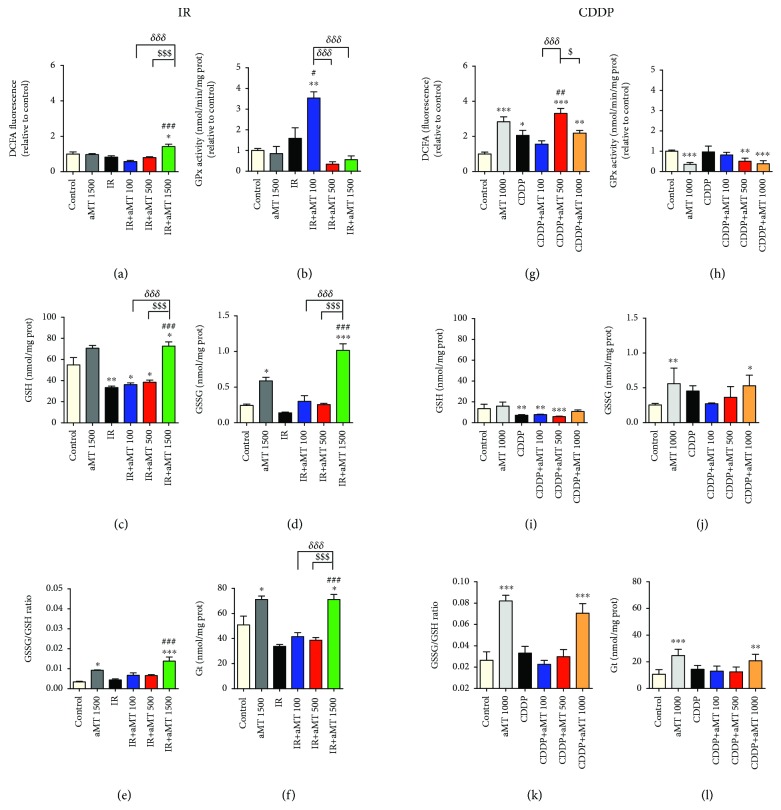
Combined treatment with melatonin and IR or CDDP increases oxidative stress in the HNSCC cell line Cal-27. Measurements of intracellular ROS levels by fluorometry after staining with the DCF fluorescent probe (a, g), GPx activity (b, h), content of GSH (c, i) and GSSG (d, j), GSSG/GSH ratio (e, k), and total glutathione (Gt) (f, l) in cells exposed to IR or CDDP, respectively. Treatment groups include the control (vehicle), IR (8 Gy), CDDP 10 *μ*M, melatonin (aMT) 1000 or 1500 *μ*M, and CDDP or IR plus aMT 100, 500, 1000, or 1500 *μ*M. *n* = 6 per group. Data are presented as mean ± SEM. ^∗^*P*<.05, ^∗∗^*P*<.01, and ^∗∗∗^*P*<.001 vs. the control; ^#^*P*<.05, ^##^*P*<.01, and ^###^*P*<.001 vs. the IR- or CDDP-treated group; *^δ^P*<.05, *^δδ^P*<.01, and *^δδδ^P*<.001 vs. IR+aMT 100; ^$^*P*<.05, ^$$^*P* < .01, and ^$$$^*P* < .001 vs. IR+aMT 500.

To determine whether mitochondrial changes correlate with an increase in ROS, we measured ROS generation intensity using the DCFH-DA probe (Figure 4(a)). We observed a significant increase in intracellular ROS levels at melatonin 1500 *μ*M in irradiated cells and, consequently, an increase in the GSSG/GSH ratio (Figures 4(c)–4(e)). Moreover, melatonin 500 and 1000 *μ*M resulted in a sharper increase in ROS production in cells exposed to CDDP as compared to irradiation (Figure 4(g)). However, the increase in the GSSG/GSH ratio was only observed at 1000 *μ*M (Figures 4(i)–4(k)), indicating that high doses of melatonin increased glutathione synthesis (Figures 4(f) and 4(l)). A parallel decrease in GPx activity was observed at the highest concentration of melatonin, especially in cells treated with CDDP (Figure 4(h)). However, at melatonin 100 *μ*M combined with irradiation, we observed an increase in GPx activity (Figure 4(b)). These results are consistent with the lower levels of ROS observed at melatonin 100 *μ*M as compared to 1500 *μ*M. These data suggest that mitochondria cause a melatonin-induced ROS response in cancer cells which enhances the cytotoxic effects of irradiation and CDDP. Furthermore, despite being known to be a strong antioxidant, previous studies have shown that melatonin increases ROS production in tumor cells [18]. Thus, melatonin induces apoptosis in HNSCC by generating intracellular ROS and activating the mitochondrial pathway, thus increasing the effect of irradiation and CDDP.

## 4. Discussion

Radio- and chemotherapeutic resistance remains the major obstacle to successful cytotoxic therapy for human cancers. After the failure of the usual treatments, following chemo- or radiotherapy, even more distressing situations occur. Therefore, finding a coadjuvant treatment to suppress or reduce this resistance would represent a major advance for both patients and the health system as a whole. On the other hand, the treatments currently used usually present a high degree of toxicity in healthy cells. In this study, we demonstrate that melatonin significantly affects sensitivity to irradiation and CDDP in HNSCC, as reflected by reduced cell proliferation and clonogenicity, as well as apoptosis induction. Although the antitumor activity of melatonin has been reported elsewhere [27–29], the potential mechanisms involved remain unclear. Despite all the studies carried out, there are a disconcertingly large number of possible mechanisms which could explain the oncostatic effects of melatonin, involving almost as many mechanisms as tumor types. This suggests that only epiphenomena of an as yet unknown underlying mechanism of melatonin have been observed [29, 30].

In this study, we not only demonstrate the role of melatonin in improving the effects of irradiation and CDDP antitumor treatment but also, more importantly, describe the possible mechanisms involved in combined treatments which enhance their anticancer properties.

Cancer cells display increased resistance to regulated cell death, often due to alterations in the mitochondrial control of this process [31]. It has been suggested that the ability of most cancer cells to flexibly rewire their mitochondrial metabolism underlies multiple instances of chemoresistance [9]. Moreover, as melatonin has recently been shown to increase neural stem cell differentiation due to increased mitochondrial function [32], a similar mechanism could, in our view, occur in tumor cells. We therefore hypothesize that melatonin raises ROS production levels by increasing mitochondrial function in tumor cells. Consequently, cellular differentiation is expected to be caused by a change in cell metabolism, thereby increasing tumor sensitivity to other drugs. In this study, we provide, for the first time, evidence that the increase in irradiation and CDDP cytotoxicity caused by melatonin is partly due to enhanced mitochondrial function.

Our results show that melatonin-induced apoptosis coincides with a significant upregulation of the proapoptotic protein Bax and a downregulation of the antiapoptotic protein Bcl-2 on HNSCC. Bcl-2 overexpression is often associated with considerable cancer cell resistance to apoptosis [33]. When the Bax/Bcl-2 ratio increases, the mitochondrial permeability transition pore opens which, in turn, releases apoptogenic mitochondrial proteins to activate caspases which induce cell apoptosis [34]. Interestingly, the combined melatonin and cisplatin treatment had higher cytotoxicity than the combined treatment with irradiation. The main bulk of irradiated cells may take longer to enter apoptosis than those treated with CDDP. Other authors, such as Mirzayans et al. [35], have made a similar observation that different cancer cell lines such as HCT116, MCF7, and AT12 are markedly refractory to apoptosis in response to ionizing radiation. In this regard, it is possible to broadly classify cell death mechanisms into two classes: those occurring relatively soon after irradiation and before cell division leading to early cell death and those occurring comparatively late or after division leading to late cell death. The vast majority of proliferating normal and tumor cells die after a relatively long period following irradiation, usually after attempting mitosis one or more times [36]. As a result, alterations in a particular gene may dramatically alter the levels of radiation-induced apoptosis, without changing the overall ability of the cell to survive [36]. In this case, cells die regardless of whether apoptosis is subsequently induced. On the other hand, the cytotoxic effect is dependent on drug concentrations, time of exposure, and time after exposure [37]. Generally, CDDP induces cell cycle arrest or apoptosis when administered at lower concentrations and induces necrosis at higher concentrations, although the effect differs between cell lines. Since CDDP exposure induces mitochondrial impairment and subsequently promotes cell death [38], high levels of mitochondrial metabolic activity are expected to enhance cisplatin cytotoxicity in cancer cells.

In order to mechanistically explain our findings, we investigated the effect of the combined treatments on mitochondrial respiration. We found that melatonin combined with irradiation or CDDP increased ETS capacity and ATP production. During late-stage apoptosis, ATP levels declined, mainly due to mitochondrial function loss and reduced consumption by ATP-dependent proteases [39]. In line with this finding, we observed a decrease in ATP levels following the combined treatment with irradiation and melatonin 1500 *μ*M as compared to the combined treatment with melatonin 500 *μ*M. These results are consistent with the increase in apoptosis at melatonin 1500 *μ*M alone or combined with irradiation. However, the combined treatment with melatonin and CDDP was more toxic than that with irradiation. The occurrence of ATP deprivation in all types of cell death suggests that energy metabolism may play a critical role in cancer cell survival under stress conditions. Thus, we demonstrate that the strong antitumor effect of irradiation or CDDP combined with melatonin is partly due to enhanced mitochondrial function.

Mitochondria also contain other molecules such as mtDNA that can act as extracellular danger signals. In fact, head and neck tumor cells lacking mtDNA become cisplatin resistant [40]. Our results show that melatonin increases mitochondrial mass and mtDNA copy number in a dose-dependent manner and raises sensitivity to irradiation and CDDP.

The release of mtDNA promotes the secretion of type I interferon by malignant cells, which is necessary for the activation of optimal anticancer immune responses following chemo- and radiation therapy [41]. Thus, mtDNA also acts as a danger signal [42] linking intracellular stress responses to the preservation of extracellular homeostasis [43].

In addition, autophagy is one of the principal mechanisms involved in controlling cellular homeostasis [44, 45]. Although there is evidence to suggest that autophagy has a prosurvival function, excessive autophagy may lead to cell death, a process morphologically distinct from apoptosis [46]. Moreover, autophagy-deficient malignant cells, which succumb to *in vivo* chemotherapy and radiation therapy, lose their ability to drive anticancer immunity [9]. We detected increased expression of ATG12-ATG5, which plays a critical role in the biogenesis and elongation of the autophagosomal membrane following treatment with high concentrations of melatonin combined with irradiation or CDDP. However, NIX, which is required for the selective mitophagy-dependent elimination of mitochondria [47], only increased with high concentrations of melatonin combined with CDDP. The role of mitophagy in cancers is controversial. Mitophagy can facilitate survival through adaptation to stress, and mitophagic defects also promote metastatic dissemination [48], most likely due to moderate overproduction of ROS [49], which activate several signal transduction cascades associated with metastatic dissemination [49]. Conversely, under severe oxidative stress conditions, ROS de facto inhibit metastatic dissemination, most likely as a direct consequence of increased apoptosis [50, 51].

Therefore, given that mitochondria are the main source of cellular ROS, we hypothesize that melatonin enhances the cytotoxic effects of irradiation and CDDP caused by increased ROS production. Our study shows that melatonin significantly increases intracellular ROS in cells exposed to irradiation or CDDP. However, ROS production levels increased following the combined treatment with CDDP as compared to treatment with irradiation. Other research, which reinforces our conclusions, has found that the ROS-scavenging enzyme expression increases in CDDP-resistant cancer cells as compared to normal cells and that mitochondrial dysfunction may bestow resistance on CDDP due to the absence of or reduction in mitochondrial ROS responses [26].

The accumulation of intracellular ROS, which damages organelle proteins, enzymes, and membranes, eventually activates apoptosis signaling pathways [52]. ROS can also cause cell death either directly or through activation of intracellular proapoptotic pathways [52]. Besides triggering apoptosis, oxidative stress can promote permanent proliferative arrest known as cellular senescence [53].

Increasing oxidative stress-targeting mitochondria is therefore a novel therapeutic strategy for selectively killing cancer cells. Our results clearly indicate that the potentiation of the cytotoxic and proapoptotic effects of irradiation and CDDP by melatonin is mediated, at least partially, by the activation of mitochondrial function and subsequent overproduction of ROS *in vitro* in a dose-dependent manner. Our data suggest that there is a correlation between the cell content of melatonin and its apoptotic effects, thus supporting the notion that high concentrations of melatonin in cancer cells are required to enhance the cytotoxic effect of irradiation or CDDP.

A major limitation in the use of irradiation for therapeutic purposes is the development of side effects. Previous studies have shown that melatonin not only enhances the oncostatic effects of radio- and/or chemotherapy on tumor cells but also protects normal cells against the adverse effects of these treatments [18, 54–56]. We have patented a melatonin gel to prevent mucositis, which has completed a phase II clinical trial (EudraCT number: 2015-001534-13) in 80 patients with head and neck cancer, and have demonstrated that melatonin protects oral mucosa against the side effects of radiotherapy. All these data show that melatonin constitutes an innovative adjuvant strategy in the treatment of cancer.

## 5. Conclusions

Our study suggests that high doses of melatonin sensitize cancer cells to CDDP and irradiation by enhancing their mitochondrial function. Melatonin is a ROS inducer in HNSCC, and the accumulation of intracellular ROS plays an upstream role in mitochondria-mediated apoptosis. Our findings indicate that melatonin has great potential not only in augmenting radio- and chemosensitivity to cisplatin and other treatments but also in reducing toxicity caused by radio- and chemotherapeutic agents in cancer patients. Our study could provide a basis and guidelines for the application of treatment with melatonin combined with radiotherapy or CDDP or other chemotherapeutic agents to improve therapeutic efficiency for cancers, especially for head and neck squamous cell carcinoma.

## Data Availability

The datasets generated during and/or analyzed during the current study are available from the corresponding author on reasonable request.
